# Bacteriology

**Published:** 1907-09

**Authors:** D. D. Crews

**Affiliations:** Fort Myers, Fla.


### BACTERIOLOGY.

By Dr. D. D. Crews, Fort Myers, Fla.

It is not the intention of the essayist to present anything new or entirely original to the all important subject of Bacteriology, but to touch lightly on the relation that it bears to our profession.

No specialist of medicine is confined to an area or part of the body which affords a much better culture, and I might say greater variety of bacteria than is the dentist. Is it not a fact that the oral cavity is the media through which a large per cent. of the diseases of the body are directly transmitted, for example: Diphtheria, Tuberculosis, Pneumonia, Cholera, etc., not to mention the other diseases that may be caused indirectly from bacteria found in the mouth?

The importance of the subject being seen, hence the necessity of a mastery of the fundamental principles of the science by the Dental Surgeon. No department of dental science has received less consideration from the profession at large than has that of Bacteriology. Only in recent years have the faculties of our colleges recognized the importance of this branch of medicine and placed it in the curriculum, and now our dental schools have and operate their own bacteriological laboratories.

It is a study too often viewed by the college student and practitioner as theoretical and of no practical value to the busy dentist. It is true that the average practitioner is so busily engaged in curing rather than preventing dental decay, that he finds but little time to devote to scientific investigation. and research, however, it is the belief of the writer that in proportion as the dental practitioner becomes acquainted with and versed in the science of Bacteriology, in the same proportion will he be enabled to render to his clientele a service superior to that of the unlearned in these lines (other qualifications being equal).

But let us consider for a few brief moments the practical side of the question and see if we can not call into our daily practice some principles and practices which will tend to lessen the number of bacteria in the mouths of our patients. We owe it to our clientele to have our office and environment to appear clean and free from bacteria, as well as our own personnel. Our instruments should be thoroughly cleansed and then sterilized, by chemicals in solution, or by boiling or whatever process is most available and efficient.

There are two factors in preventing and lessening the bactercidal influences of the oral cavity, viz.: the dentist and patient. First.—The dentist's part will consist chiefly in properly inserting, contouring and finishing fillings, treating pathological conditions existing, such as putrescent pulps, abscessed teeth, pyorrhea alveolaris, correcting irregularities, extracting roots, removing calculus, polishing teeth, etc. This in a brief manner will constitute his operative work. The other part of his service will consist in instructing his patients in the proper care of their teeth and adjacent parts. This instruction can be supplemented largely by keeping for distribution among patients a small treatise on the care of the teeth.

Second.—The patient's part will consist in keeping the mouth clean, this they are to do by the use of tooth picks and floss silk followed by antiseptic washes and dentrifices. Special attention and instruction should be given the patient as regards the form of tooth brush as well as the manipulation of same. It is the observation of the writer that at least 75 per cent. of the brushes used by our patients are incorrect in form.

The dentist is constantly asked what dentrifice would he recommend for keeping the teeth clean. In answnr to this query I would recommend at least three, a good paste for constant use, a powder for occasional use and some liquid antiseptic wash to be used before retiring and the first thing on arising. The form of liquid antiseptics employed should be governed by the case in hand; for patients with an acid reaction of the secretions and pregnant women I would recommend the use of a mouth wash which is antiseptic and decidedly alkaline, as Philip's Milk of Magnesium or Glyco-Thymoline.

The above enumerated are a few of the many ways in which the dentist can help to bring about a more ideal condition of the oral cavity. Dentistry like medicine is advancing along scientific lines, and what the analytic chemist is to the soil in analyzing and finding out the composition and elements needed, so in the near future must the dentist be able to analyze from time to time the secretions of the mouth and prescribe accordingly.

